# Blending online therapy into regular face-to-face therapy for depression: content, ratio and preconditions according to patients and therapists using a Delphi study

**DOI:** 10.1186/s12888-014-0355-z

**Published:** 2014-12-14

**Authors:** Rosalie van der Vaart, Marjon Witting, Heleen Riper, Lisa Kooistra, Ernst T Bohlmeijer, Lisette JEWC van Gemert-Pijnen

**Affiliations:** Department of Psychology, Health & Technology, University of Twente, Enschede, the Netherlands; Health, Medical and Neuropsychology Unit, Leiden University, Leiden, the Netherlands; Lectorate Community Care & Youth, Saxion University of Applied Sciences, Enschede, the Netherlands; Department of Clinical Psychology, VU University Amsterdam, Amsterdam, the Netherlands

## Abstract

**Background:**

Blending online modules into face-to-face therapy offers perspectives to enhance patient self-management and to increase the (cost-)effectiveness of therapy, while still providing the support patients need. The aim of this study was to outline optimal usage of blended care for depression, according to patients and therapists.

**Methods:**

A Delphi method was used to find consensus on suitable blended protocols (content, sequence and ratio). Phase 1 was an explorative phase, conducted in two rounds of online questionnaires, in which patients’ and therapists’ preferences and opinions about online psychotherapy were surveyed. In phase 2, data from phase 1 was used in face-to-face interviews with therapists to investigate how blended therapy protocols could be set up and what essential preconditions would be.

**Results:**

Twelve therapists and nine patients completed the surveys. Blended therapy was positively perceived among all respondents, especially to enhance the self-management of patients. According to most respondents, practical therapy components (assignments, diaries and psycho-education) may be provided via online modules, while process-related components (introduction, evaluation and discussing thoughts and feelings), should be supported face-to-face. The preferred blend of online and face-to-face sessions differs between therapists and patients; most therapists prefer 75% face-to-face sessions, most patients 50 to 60%. The interviews showed that tailoring treatment to individual patients is essential in secondary mental health care, due to the complexity of their problems. The amount and ratio of online modules needs to be adjusted according to the patient’s problems, skills and characteristics. Therapists themselves should also develop skills to integrate online and face-to-face sessions.

**Conclusions:**

Blending online and face-to-face sessions in an integrated depression therapy is viewed as a positive innovation by patients and therapists. Following a standard blended protocol, however, would be difficult in secondary mental health care. A database of online modules could provide flexibility to tailor treatment to individual patients, which asks motivation and skills of both patients and therapists. Further research is necessary to determine the (cost-)effectiveness of blended care, but this study provides starting points and preconditions to blend online and face-to-face sessions and create a treatment combining the best of both worlds.

## Background

Depression is one of the most commonly diagnosed mental disorders among adults, which causes a major public health problem [1]. While effective psychotherapy for depression is available, many patients do not receive appropriate treatment since it is time-consuming and costly, and mental health care institutions often do not have the capacity to provide everyone with the care they need [2,3]. Innovations in web technology offer promising perspectives to decrease these issues, by providing adequate care in a low key and efficient manner, often through online self-help or guided self-help programs. In these programs patients mostly follow a standardized psychological treatment (cognitive behavioral therapy) via the internet, with minimal feedback or support [4,5].

Recent systematic reviews and meta-analyses have shown that online psychological treatments for depression often are as effective as face-to-face psychological treatments [3,6,7]. Nevertheless, problems with adherence to the online program and translation of therapy content into daily life can be difficult for patients [8-10]. Most systematic reviews found that offering some form of support or guidance during the online treatment increases its effectiveness and is associated with higher levels of completion [3,6,11]. Studies on the experiences of patients with online therapy also show that personal feedback and support are perceived as positive among patients, to optimally use the program and to keep them motivated [12-14].

Up until now, the largest supply of eHealth interventions for common mental disorders focuses on the general community and to a lesser extent on primary care, reaching people who suffer from mild to moderate depressive symptoms [3,6,15]. The availability of eHealth interventions as an integrated part of routine practice, however, is still scarce. While standalone eHealth interventions could also be beneficial in secondary health care, patients with more severe symptoms of depression may need different programs or a more intensive guidance in their online therapy in order to reach recovery [4,11,16]. While recent studies have shown that adding online sessions or modules to regular face-to-face psychotherapy can increase the adherence and effectiveness of a patients’ treatment, it also increases costs and workload among professionals [17-19]. To overcome these drawbacks, adopting a combination of online and face-to-face therapy, in which online sessions replace or substitute some (parts) of the sessions with a health professional, could offer patients the best of both worlds [13,20]. Patients would then receive a blend of face-to-face sessions with a health professional and modules in an online program which they follow independently, while the actual duration of therapy stays equal or could become shorter. Presumably, this blend of online and face-to-face sessions could increase the fit between the severity of a patient’s complaints and the intensity of the treatment; patients who are better able to self-manage their mental health could receive less face-to-face sessions, patients that need more guidance and support could receive more face-to-face sessions compared to online sessions.

While mental health care institutions are increasingly introducing this form of therapy in their services, studies on blended mental health care are scarce. Evidence-based rationales for the content and ratio of the combined online and face-to-face sessions are, therefore, lacking [7,21]. The optimal level of support, the nature of the support, and essential preconditions to make blended care acceptable and cost-effective have not been studied as well [11,10,22]. In order to thoroughly develop and implement such an innovation it is key to use a structured method which involves both therapists and patients. Van Gemert et al. [23] created the CeHRes-roadmap for development and implementation of e-Health interventions. This roadmap uses a bottom-up process to incorporate the context of a particular health care setting and the values of all stakeholders and end-users of an intervention, to improve the implementation and impact of eHealth technologies. The involvement of end-users in the early stages of development is crucial to create commitment and to determine the requirements for design.

Following this roadmap, the aim of this study was to gain (1) insight into which treatment components are most suitable for online and/or face-to-face sessions, (2) the sequence and ratio the two forms of therapy should be combined in, and (3) the essential points of interest that should be considered when implementing blended care for depression in secondary mental health care (i.e. health care services provided by psychiatrists or clinical psychologists). Needs and preferences of both patients and therapists were studied to gain insight into the optimal use of blended therapy in mental health care for depression.

## Methods

A Delphi method was used [24], which aimed to find consensus on suited protocols, concerning content, sequence and ratio to blend online and face-to-face therapy for depression. Phase 1 was an explorative phase, conducted in two rounds, in which patients’ and therapists’ preferences and opinions on online psychotherapy were surveyed using online questionnaires. The main aim of this phase was to investigate which components of the therapy could be best executed online and face-to-face, and how online and face-to-face sessions should be distributed over the whole therapy plan. In phase 2, data from phase 1 was translated to clinical practice, using interviews with therapists to investigate more in depth how actual blended therapy protocols could be set up and what essential preconditions would be to make this form of therapy feasible in secondary mental health care practice.

### Recruitment of participants

Three mental health care institutions with interest in blended therapy, from different regions of the Netherlands, were involved in the study. Two institutions were already offering patients blended psychotherapy and one institution intended to provide this in the near future. From these institutions both therapists and patients were asked to participate in the study. The department manager of each institution invited therapists that currently treated patients with major depression, through an information letter explaining the purpose of the study and what participation would entail. To recruit patients, each therapist was asked to invite one of his/her patients to participate, with an information letter. Upon participation, all interested therapists and patients were asked to fill out an informed consent, including their e-mail address. Participants were approached in phase 1 via e-mail to complete the (anonymous) questionnaires by following a secured web link. In phase 2, all the included therapists were asked to participate in individual interviews, via an e-mail containing an information letter. According to national regulations in the Netherlands the study did not need approval of the ethical review board. While our research includes human participants (patients and therapists) in a medical setting, it does not impose upon physical or psychological integrity. The Central Committee on Research Involving Human Subjects [CCMO] states that: “In general, research with human subjects only falls under the Medical Research (Human Subjects) Act [WMO] if there is an infringement of the (psychological) integrity of the subject” (www.ccmo.nl/en/medical-scientific-research-and-the-wmo; www.ccmo.nl/en/questionnaire-research).

### Phase 1: explorative and confirmative surveys

The survey in round 1 started with asking background information of the participants (age, gender, educational level, and experience with online therapy). Then, participants were asked about possible benefits and drawbacks they experienced or would perceive from online or blended therapy. They were asked to use a text box and to give as much answers as they could think of. Subsequently, participants’ preference for online or face-to-face contact was explored related to the main components of a standardized treatment: intake, information/education, assignments, diaries, and evaluation. After every main topic a text box was provided for elaboration/explanation and respondents were encouraged to use this space. Finally, participants were asked what their ideal distribution of online and face-to-face sessions among the whole therapy would be in percentages, ranging from 10% online/90% face-to-face to 90% online/10% face-to-face (nine options in total).

Results from round 1 were analyzed by quantifying scores on each item, by calculating percentages of respondents’ answers and by evaluating the consensus in the answers. Based on the consensus, the items were reformulated and used as input for round 2 [25].

In round 2 a second survey was sent to the same respondents, which started off with a small summary of the previous round. In the first items all the benefits and drawbacks from round 1 were used as statement items to gain consensus on the attitudes of respondents concerning online and blended therapy. The statements were arranged in main topics that could be derived from round 1, using a thematic analysis. The themes addressed possible changes in self-management of the patient, changes in the patient-provider relationship, changes in face-to-face sessions and diverse possible drawbacks concerning online therapy. Participants could express their opinion on the statements using a Likert scale ranging from “totally disagree” (1) to “totally agree” (4). For example “I feel that online psychotherapy is impersonal”. Subsequently, items on the preferred mode of delivery of therapy components were used again, in a different format. Participants were asked if they agreed with statements that could be formulated from the answers in round 1. For example, if in round 1 a small majority indicated to be in favor of providing homework assignments online, the statement would be “I prefer providing homework assignments online”, using answer categories ranging from “totally disagree” (1) to “totally agree”(4). This way, we were able to gain an overview of what treatment components should be done online and face-to-face, according to the majority of the participants. For all statements, there was no “neutral” response option, because we wanted to encourage participants to agree or disagree. If participants did not want or could not answer the question they could skip the item without answering it. In the last section the ratio in percentages of online and face-to-face sessions was explored using the data from round 1. Based on these answers, in round 2, five options were given to the respondents, ranging from 25% online/75% face-to-face to 75% online/25% face-to-face.

### Phase 2: interviews to translate results into practice

In phase 2 the same group of therapists from phase 1 was invited for an interview. If therapists were willing to participate, an interview at the therapists’ institution was planned by e-mail or telephone. In the interview the results from the surveys were briefly presented and the aim of the interview was explained; namely to investigate how scenarios for blended care could be best designed to be suitable for clinical practice and what preconditions would be necessary to use blended care in secondary mental health care (based on the benefits and drawbacks from round 1). The main questions were: “If you look at the current depression protocol, keeping the survey results in mind, what specific components or assignments in the therapy need face-to-face guidance, in your experience?”, “What components or assignments in the therapy can patients do independently, online?”, and “In your experience, what are essential preconditions to make blended care feasible in secondary mental health care?”.

### Data-analysis

Results from phase 1 were analyzed by quantifying scores on each item from the survey in round 2 and calculating percentages of patients and therapists who chose a certain answer on the items. In phase 2, the audiotapes of the interviews were transcribed verbatim and coded by two independent researchers. Using explorative data analysis, for each main question from the interview scheme citations were extracted and arranged into themes and subthemes. Subsequently, these categories were discussed between the two researchers, until consensus about the final categories was reached. Next, the first researcher (RV) examined the raw data again to ensure the robustness of the analytical process and to confirm that all the data was indeed reflected in the coding [26]. During this process, only participant numbers were used to protect the anonymity of the participants.

## Results

### Phase 1

#### Respondents

Twelve therapists of which eight female and four male completed the survey in both round 1 and 2. Their mean age was 45 years old, with a range from 28 to 60. Six of them already had previous experience with online treatment for depression. Nine patients, three female and six male, completed the survey in round 1, with a mean age of 37 and a range from 23 to 56. The educational level of these respondents ranged from elementary school (*n* = 1) to bachelor’s degree (*n* = 1). One of the patients was currently following online therapy; the others did not have any experience with online treatment. In round 2, eight out of nine patients completed the survey, two female and six male with a mean age of 37 years.

#### Attitudes on online treatment by patients and therapists

The results in Table 1 show attitudes of therapists and patients regarding online treatment. It can be seen that online sessions are positively perceived, especially to enhance the self-management of patients. The benefits most agreed on, related to self-management were: the convenience to always have access to the online platform, the opportunity to provide the patient with more responsibility for his/her own therapy, and incorporating the therapy more into the daily life of the patient. Furthermore, several practical benefits were mentioned: the ability to perform assignments in a patient’s own time and pace and the deduction of travel time.

**Table 1:** **Benefits and drawbacks of blended (online) therapy for depression according to therapists and patients (phase 1)**

	**Therapists (n = 12)**		**Patients (n = 8)**	
**Statements**	**(totally) agree** ^**a**^	**(totally) disagree** ^**a**^	**(totally) agree** ^**a**^	**(totally) disagree** ^**a**^
*Self-management*				
Convenient to always have access to therapy content	92 (11)	8 (1)	100 (8)	0 (0)
Encourages patients to take more responsibility for (succeeding of) therapy	83 (10)	17 (2)	100 (8)	0 (0)
Therapy blends more into the patients’ home/private situation	92 (11)	8 (1)	88 (7)	13 (1)
Sessions can be completed in own time	92 (11)	0 (0)	80 (6)	13 (1)
Sessions can be completed in own pace	83 (10)	17 (2)	80 (6)	25 (2)
Patients have to travel less	67 (8)	25 (3)	80 (6)	13 (1)
*Patient-provider relationship*				
Ability to see a patient/therapist face-to-face	83 (10)	0 (0)	100 (8)	0 (0)
Therapy structure becomes more transparent	58 (7)	17 (2)	50 (4)	38 (3)
Could provide better tailoring to the individual patient	33 (4)	17 (2)	88 (7)	13 (1)
Patient-therapist bonding could weaken	25 (3)	50 (6)	50 (4)	50 (4)
Using online sessions is too impersonal	0 (0)	67 (8)	13 (1)	50 (4)
*Changes in face-to-face sessions*				
Face-to-face sessions can be optimally used, due to preparation in the online environment	75 (9)	8 (1)	88 (7)	13 (1)
Difficulties or indistinct matters could be more difficult to discuss	25 (3)	58 (7)	50 (4)	50 (4)
*Possible drawbacks*				
It is not suitable for every patient	92 (11)	0 (0)	88 (7)	13 (1)
It could cause interpretation problems due to the lack of non-verbal communication	75 (9)	0 (0)	80 (6)	25 (2)
Therapists might need to invest much time to read all the online assignments	33 (4)	42 (5)	25 (2)	63 (5)
Patients could misuse or overuse the online environment	25 (3)	58 (7)	50 (4)	50 (4)
Personally, I lack the Internet skills	0 (0)	67 (8)	0 (0)	88 (7)

^a^% (n); answer categories were: (1) totally agree; (2) agree; (3) disagree; (4) totally disagree; blank responses are not shown in Table.

Related to the patient-provider relationship, all patients and therapists in our sample felt that they had the need to see their patient or therapist face-to-face. Most respondents did not agree with the statement that online therapy would be too impersonal, but opinions varied with respect to the statement that the use of online sessions could weaken the patient-therapist bonding. According to many respondents, it could make the therapy more transparent because patients can exactly follow the structure of the therapy and the sequence of the sessions.

Concerning changes in regular face-to-face therapy, most respondents agreed that it could improve the usefulness of the sessions, because they could better prepare the face-to-face sessions, using the input from the online sessions.

The largest drawbacks that were agreed on was that online therapy would not be suitable for every patient and that problems with interpretation could occur due to a lack of non-verbal communication. All respondents, however, felt that they would have enough internet skills to properly use online therapy modules.

#### Preferences for online and face-to-face content and process

Table 2 shows the preferences for online and face-to-face content concerning the main components of the therapy. The only item on which initial consensus was found concerned the introduction of the treatment; when getting to know one and other as therapist and patient all respondents were in favor of face-to-face contact. Furthermore, concerning the process of the treatment face-to-face contact is much preferred to ask questions, and to share thoughts, feelings and difficulties with assignments. Practical components of treatment, such as assignments, psycho-education and diaries could be performed online according to most respondents. Preferences of patients and therapists were highly similar on these matters.

**Table 2:** **Suitable therapy content for online and face-to-face session according to therapists and patients (phase 1)**

	**Therapists (n = 12); % (n)** ^**a**^		**Patients (n = 8); % (n)** ^**a**^	
Mode of delivery	Online	Face-to-face	Online	Face-to-face
Content				
Treatment introduction	0 (0)	100 (12)	0 (0)	100 (8)
Treatment evaluation	17 (2)	67 (8)	0 (0)	88 (7)
Assignments	75 (9)	8 (1)	63 (5)	38 (3)
Psycho-education	100 (12)	0 (0)	75 (6)	25 (2)
Mood and activity diaries	100 (12)	0 (0)	88 (7)	13 (1)
Process				
Asking questions about assignments	8 (1)	83 (10)	25 (2)	75 (6)
Expressing thoughts, feelings and difficulties on assignments	33 (4)	67 (8)	0 (0)	88 (7)
Expressing thoughts, feelings and difficulties on diary content	17 (2)	83 (10)	13 (1)	75 (6)
Reminders to complete assignments and diaries	75 (9)	17 (2)	88 (7)	0 (0)

^a^blank responses are not shown in Table.

#### Distribution of online and face-to-face therapy in percentages

Table 3 shows the ideal ratio of online and face-to-face sessions in the whole course of therapy, according to the respondents. What stands out is that a large part of therapists wants to see their patients in person for the main part of the therapy (75% face-to-face sessions), Patients are, overall, a little less conservative and would like to perform half or slightly more of the therapy sessions in an online environment (50 to 60%).

**Table 3:** **Preferred ratio of online and face-to-face sessions in depression therapy, according to therapists and patients (phase 1)**

**Ratio in percentages**	**Therapists; % (n)**	**Patients; % (n)**
75% face-to-face/25% online	33 (4)	0 (0)
60% face-to-face/40% online	8 (1)	13 (1)
50% face-to-face/50% online	25 (3)	38 (3)
40% face-to-face/60% online	17 (2)	38 (3)
25% face-to-face/75% online	0 (0)	0 (0)

### Phase 2

#### Respondents

Of the twelve therapists in round 1, nine agreed to participate in an interview. The others were not able to participate due to time constraints. Three men and six women were interviewed, with a mean age of 45, ranging from 28 to 60.

#### Applying blended care in clinical practice

In the analysis of the main questions, related to components that could be performed independently and components that would need face-to-face support, all respondents showed difficulty in talking about this in a general sense. When asked about what “a typical patient” could do online or not, all interviewees answered that a typical patient does not exist in secondary mental health care and that protocols cannot be followed exactly as they are provided.

*“The content of every therapy is different. ‘The average patient’…we do not see average patients…” (Female, 60 years old).*

*“A therapy is different every time, and the assignments that I give are different every time as well. So, it is very difficult for me to say: this assignment should be done here in the process, this one there, or these sessions there. Therefore, it is very complicated for me to think about what can be entirely done online”. (Male, 54 years old).*

All therapists emphasized that patients in secondary mental health care, suffering from major depression, have severe and complicated problems. Patients often suffer from personality disorders as well, and they often experience problems in several areas of life (personal relationships, work, or financially). This complexity in problems asks for a flexible treatment, which often hinders following a strictly protocolized therapy. Based on these reactions, it became clear that there is a need for flexible, non-standardized, scenarios for blended therapies, aimed at individual patients. Therefore, developing standard blended protocols with a pre-set plan for routing and content of every session was not feasible in the interviews, and the focus of the interviews often shifted more to the preconditions that are necessary to make blended care usable in clinical practice and to support tailoring of blended protocols.

Predominantly, the use of face-to-face sessions is crucial; to create commitment, to activate and motivate patients, to monitor patients and to keep up the pace of the treatment. In every part of the treatment, patients need extensive guidance in translating the theory and assignments of the therapy into their own life and to become activated in a way that matches their abilities.

*“We sometimes use online modules, to monitor activities or thoughts. But we never say: “Well, you could do this step on your own, just read it through and finish it by yourself”. Then they just won’t do it. Not because of unwillingness, but we treat many people that are just not like that”. (Male, 54 years old).*

The other preconditions that were mentioned are shown in Table 4, including mentioned practical steps to fulfill these preconditions. According to the therapists, taking patients’ characteristics into account is essential in order to match the online therapy with the individual patient. Meta-communication about the form of therapy, how the content will be presented, and for what reason, will help to explain the rationale of the therapy. To show how useful online tools can be, patients can start filling in intake forms right away and start their treatment.

**Table 4:** **Preconditions to use blended care for depression in secondary care, according to therapists (n = 9) (phase 2)**

**Preconditions**	**Practical steps to incorporate this precondition**
Flexibility in the online program	Blended therapy should be tailored to the individual patient, based on individual needs.
	Ratio of online and offline sessions should be chosen during the therapy.
	Content of online sessions should be flexible, by working with separate online assignments or modules.
Take patient characteristics into account	Involve patients into the choice for (and ratio of) blended therapy.
	Consider patients’:
	▪ Co-morbidity (rule out crises; depression should be the main focus of therapy)
	▪ Needs and motivation
	▪ Intelligence (can they express themselves in writing)
	▪ Skills (can they use a computer)
	▪ Personality (self-management, discipline)
Meta-communication with patients on blended care	Tell them why you work with blended therapy.
	Show them how the program works.
	Tell them it is important to finish the whole therapy, even though they might feel better half way through.
	Tell them it is essential that they do the work and that the therapist only plays a supporting role.
Use online registration and intake modules	The patient knows that online technology will be used in therapy from the start.
	Patients are offered help immediately, they can start their therapy (intake) without waiting for an available therapist.
	Emphasis is on patient self-management from the start of the therapy.
Use innovations that technology offers beneficially	Do not just copy a face-to-face protocol in an online program.
	Alternate reading texts with active assignments.
	Use animated examples and videos.
	Use persuasive technology to motivate patients and to support discipline.
	Create a database with varying online tools and modules that can be applied as suited on each individual patient.
Train and educate therapists	Therapists should be motivated to work with blended therapy.
	Workflow and time investment will change.
	Technical and practical skills are needed (how to support patients, when and how to provide online feedback).
	Balance in “letting patients go” and stimulating and supporting them.
Implementation should be initiated and stimulated from the management	Therapists need to be trained which costs time and effort, this should be accommodated by the management.

*“I would like to use many more online tools at the start of the treatment. To give patients the idea that something is happening and that we are helping them, while they do the largest part of the work themselves. If you could get a patient to work on something, they feel that they are doing something to get better. Then the empowerment of the patient grows. Patients feel much more competent if they can start something right away”. (Male, 59 years old)*

In order to encourage patients to use the program, to support usability, and to increase the discipline to keep using the program, innovations in technology should be used. According to the therapists, animations, videos and expressive examples could help patients understand the theory and assignments, without reading large amounts of texts. Online feedback, reminders and stimuli (such as games, collecting points) might encourage the motivation and discipline of patients to stay in therapy and to fulfill the assignments. Also, the therapists would highly appreciate a large database of available online assignments. This way, the online components would not be fixed and protocolled, but therapists could shape and fill in the online sessions themselves, according to the patients’ needs and abilities.

Lastly, it is not just the patients and the technology that must be suited for online therapy; the intentions and motivations from therapists and management weigh very heavy in the succeeding of blended care.

*“My concern is not to get patients motivated to use online modules, but to get therapists to use them. That is a much larger bump. Everything that is new is seen as more work, and everyone is already loaded with work and doesn’t want more work. I’m afraid that pressure from the management is the only way to get therapists to work with it”. (Female, 28 years old)*

## Discussion

This study explored the possibility to blend online therapy with face-to-face therapy in the treatment of depression in secondary mental health care, taking into account attitudes, preferences and current experiences with (online) therapy of both therapists and patients. Our results show that both patients and therapists are positive about online and blended therapy and expect more benefits than drawbacks. Essential perceived benefits were a possible increase in self-management of patients, a more independent patient-therapist relationship and better preparation of face-to-face sessions. A perceived drawback of online sessions is that certain problems or matters could become more difficult to discuss, due to a lack in non-verbal communication or because patients need intensive support in dealing with their problems. Our results show that many practical parts of the therapy could be performed in an online environment, while discussing thoughts, feelings and difficulties regarding these practical parts should still be done face-to-face. This agrees with what has been previously shown in qualitative studies on the essentials for adherence and effect of online therapy [13,8]. However, a large part of our sample had no previous experience with providing or receiving online therapy. The benefits, drawbacks and preferences reported are mostly *perceived* and it could very well be that participants find it difficult to imagine what online therapy could look like and how it would be conducted. Pilot studies using both quantitative and qualitative data on experiences, feasibility and adherence should be performed to provide more information on these matters. Notably, there was a large focus on text-based online therapy among our participants, including the use of e-mail to communicate. Because online therapy is still scarce in clinical practice many therapists and patients are probably not aware of the large amount of other possibilities that technology can offer. An online intervention is not just a simple translation of a regular face-to-face therapy to an online environment [27]. Using other forms of media such as video, or gamification elements could broaden the potential of online and blended therapy for many people. However, in that case it is still essential to know what kind of modality or persuasive technology fits specific content and individual patients [28]. Future research should focus more on how to optimally employ these possibilities.

Therapists stress that patients’ characteristics should be carefully taken into account in the choice for blended care, and the online program should be flexible enough to fit a patient’s specific needs. A review by Barak et al. [29], previously showed that the satisfaction and experienced benefits and barriers of online therapy versus face-to-face therapy can differ between individuals and between target groups, depending on the severity of the depression and characteristics of the patient (internet skills, personality, self-management skills). Because of this difference among patient groups, clear scenarios of how blended care could be protocolized in clinical practice could not be gained from our interviews. Models on essential factors of human support in online treatment could provide more insight into how support can be optimally delivered to enhance adherence and satisfaction within individual blended treatments [30]. To provide therapists guidance in deciding when and for whom blended treatment will be suitable, the results of this study have been used to develop an instrument to advise when, and in what ratio, online and face-to-face sessions could be used [31,32].

Flexibility in blended programs is one of the main benefits to support patients in their treatment; notwithstanding, it demands commitment, willingness and skills of therapists [33]. They should learn how to use the online modules in a stimulating way and they should be able to fit therapy to individual patients. Also, their workflow will change, as patients are no longer seen for just one hour per week, but are monitored and supported more frequently, for smaller durations at a time. A previous meta-analysis showed that a higher intensity in therapy has the potential to increase its effectiveness [34]. Blended therapy has the potential to bring about an increased intensity and continuity of therapy, by combining a face-to-face session with online modules within one week, which could possibly decrease the length of the therapy in weeks.

Using the first steps of an eHealth intervention development roadmap [23], this study offers a helpful starting point for the implementation of blended care for depression in secondary mental health care. From systematic reviews, we found that few studies up until now investigated a blend of online sessions with face-to-face sessions. In a study by Wright et al. [21], patients were seen every week by a therapist, but half of the sessions were done independently by the patient behind a computer, at the mental health care institution. A recent study by Høifødt et al. [35] investigated the effect of a guided web-based intervention with face-to-face support, with positive results on depression and anxiety symptoms. Other studies on the combination of online sessions and personal support only investigated support through e-mail, or instant messaging (e.g. [19,36-38]). Our study provides a first exploration of how the two modes of delivery could be blended optimally, integrated as equal parts of treatment. Nevertheless, concurrent with the roadmap [23], thorough development from the start of an online intervention is inevitable for proper use and effects of eHealth in general, and also of blended care. Therefore, studies should further investigate how online treatment can be optimal user-friendly (e.g. using intuitive interfaces) and how patients can maintain motivation to complete the program (e.g. by using other forms of media) [39]. When development and implementation has been taken into account, evidently, the (cost-) effectiveness of blended therapy should be studied as well (see e.g. Kooistra et al. [22]). Besides classical randomized controlled trials, log files could provide valuable data on how patients use online modules (frequency and duration), how they rate them, and to what extent patients adhere to the program. This will provide further insight into the suitability among groups and in the effectiveness of blended therapy from a different perspective, namely from what actually happens *during* the therapy [40]. This could increase our knowledge on how to tailor blended care to individual patients, related to the severity of their symptoms and their characteristics [41,42].

Although this study adds new insights to the body of literature, our results should be interpreted with care. It is possible that the patients and therapists that participated in our study have a more positive attitude to online therapy than non-responders (self-selection bias). The results on attitudes and expectations of online therapy could therefore be overly optimistic. Furthermore, the numbers of participants in our study are small. It proved to be difficult to recruit participants for such an explorative study, since not many therapists have experience with online treatment, and therapists do not receive extra time to take part in research from their management. Still, a certain point of data-saturation was reached since many care providers mentioned the same benefits, drawbacks and preconditions. Since the limited number of participating therapists led to a small number of participating patients, our sample might not be representative for the whole population of secondary mental health care patients coping with depression, especially since it is such a diverse population. We did not ask patients to participate in phase 2. This choice was made because therapists have experience with regular protocols for depression therapy among a broad range of patients. Because a therapy will never be the same for two patients we felt that therapists could better evaluate what would be suitable components and essential preconditions to combine online and face-to-face sessions from a general perspective. Lastly, it should be noted that this study particularly focused on secondary care. Obviously online therapy and eHealth modules can provide huge benefits in primary care as well, but our data cannot simply be translated to this level of care, since it considers different problems and administers different modes of therapy delivery. Future research should study the specific needs and preferences to optimally use blended care in primary care as well.

## Conclusions

This study shows that blending online sessions with face-to-face therapy has potential in secondary mental health care. In the intake period and for practical assignments online tools could be used in a low key manner, from which the database of online modules can be built up. Still, therapists (and managers) should be motivated to incorporate this in their work processes and they should develop skills to tailor the content en ratio of online sessions or modules to the severity of a patient’s problems and personality characteristics. When both patients and therapists become more adepted to online therapy and experience the possibilities of technology, blending online sessions into face-to-face therapy could create a treatment using the best of both worlds.
